# Dercum's Disease: A Rare Disease of Painful Fatty Lumps

**DOI:** 10.7759/cureus.48615

**Published:** 2023-11-10

**Authors:** Alsadat Mosbeh, Rawan Almutairi, Abeer Albazzali

**Affiliations:** 1 Department of Dermatopathology, Farwaniya Hospital, Ministry of Health, Sabah Al Nasser, KWT; 2 Department of Dermatology and Venereolgy, Al-Azhar University, Cairo, EGY; 3 Department of Dermatology, Farwaniya Hospital, Ministry of Health, Sabah Al Nasser, KWT

**Keywords:** dercum's disease, painful subcutaneous masses, painful lumps, lipoma, adiposis dolorosa

## Abstract

Dercum's disease is a rare and poorly understood condition characterized by painful subcutaneous adipose tissue growth that can occur anywhere beneath the skin surface. We present the case of a 27-year-old man with no significant medical history who had been experiencing painful subcutaneous nodules for two years. Skin biopsy revealed the proliferation of mature adipocytes that were surrounded by fibrous septa. There are currently no treatments approved by the US Food and Drug Administration for Dercum's disease, and the effectiveness of treatments that have been attempted is variable.

## Introduction

Adiposis dolorosa, also known as Dercum's disease, is a rare and not properly understood condition characterized by painful subcutaneous adipose tissue growth that can occur anywhere beneath the skin's surface [1]. Francis Dercum, an American neurologist, described it for the first time in 1888 [2]. Many related symptoms, including fatigue, depression, dementia, and weakness, are frequently present along with the disease [1]. The disease is inherited on an autosomal dominant basis with variable penetrance, and most of the cases are sporadic [3]. The pathophysiology remains unclear [4]. The epidemiology of this condition has not yet been defined, but literature suggests a higher prevalence in postmenopausal obese females [5]. We report a case of Dercum's disease in a previously healthy male patient.

## Case presentation

We report the case of a 27-year-old man, previously in good health, who has endured two years of distressing subcutaneous nodules across his entire body, except for his head, neck, hands, and feet. The condition started with a gradual onset and was progressive. He has developed an alarming rise in the quantity of these masses. The patient also experienced paresthesia. His quality of life was significantly reduced because of painful symptoms that frequently interrupted his daily activities. Pain was refractory to analgesics and anti-inflammatory drugs. There was no history of mental disorders. The systemic review was not significant. There was no family history of obesity. He was not an alcoholic nor a smoker.

**Figure 1:**
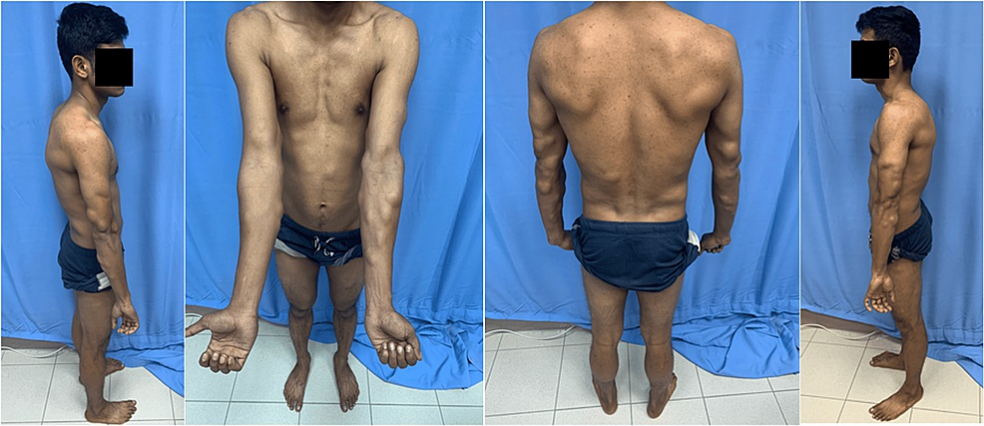
Multiple swellings scattered over the trunk, upper, and lower limbs

On examination, there were multiple soft-tissue swellings, more on the trunk and upper limbs than the lower limbs, with sparing of the face, hands, and feet (Figure 1). The swellings were painless to palpation. The patient's BMI was 24 kg/m2. Neurological evaluation was normal, and other physical findings were unremarkable.

**Figure 2:**
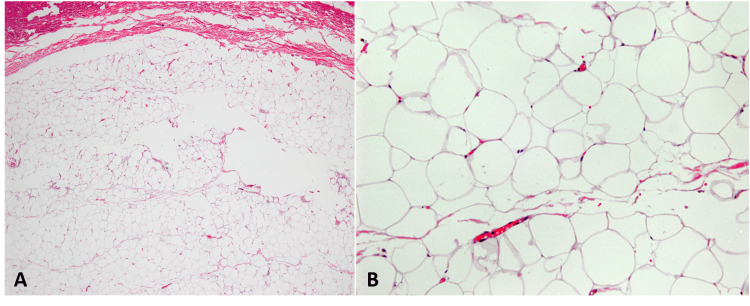
Histopathological findings of Dercum's disease A) Low-power 10X, H&E-stained biopsy of the subcutaneous mass showed fairly fibrous capsules and adipocytes with proliferating capillaries. B) High-power 20X, H&E-stained biopsy of the same patient revealed that adipocyte nuclei are small and bland, compressed at the periphery of cells. Capillaries lined with one layer of bland endothelial cells and containing RBCs.

Laboratory investigations revealed an erythrocyte sedimentation rate of 8 mm/h and a serum C-reactive protein level of 4 mg/L. Other laboratory tests, such as blood cell counts, serum electrolytes, liver function tests, serum protein electrophoresis, and lipid profiles, were normal. Skin biopsy revealed a dermal mass formed of proliferating adipocytes surrounded by fairly fibrous capsules with no atypia or mitotic figures (Figure 2). The diagnosis of Dercum's disease was reached, and after counseling the patient on the disease's nature, he preferred to continue on an oral nonopioid pain reliever (acetaminophen) without any surgical interventions. 

## Discussion

The clinical symptoms of Durcum's disease do not show a specific pattern. Many lipomas can be found in the subcutaneous tissue of the knees, back, neck, thighs, and arms of individuals with this illness. Often, the hands and face are uninvolved. Furthermore, Patients complain of paresthesia in the skin above the lipomas, which is spontaneously painful. A low threshold for pain also seems to be typical for this disease. Normal or glossy skin with dilated superficial veins may also cover lipomas [1,3,6]. Our patient presented with painful swelling on the trunk, upper and lower limbs, sparing the head, neck, hands, and feet.

Many other non-specific symptoms of Durcum's disease have been reported in the literature. It has a wide range of symptoms, such as easy bruising, sleep issues, memory loss, anxiety, depression, and difficulty concentrating; however, these symptoms are not always present. Psychiatric symptoms used to be recognized as crucial symptoms for the diagnostic criteria. These are now recognized as symptoms correlated with adiposis dolorosa, although not all individuals have them, and it is challenging to determine their exact connection to the disease [1]. Our patient didn't report any psychiatric symptoms. Other reported manifestations in patients with Dercum's disease include loss of pubic and axillary hair, myxedema, hot flushes, arterial hypertension, early congestive heart failure, cyanosis, dyspnea, and tachypnea. It remains unclear which of these are the major or minor symptoms [3]. Our patient had a normal systemic examination.

Dercum's disease is categorized into four types based on the afflicted adipose tissue's location and its relationship with lipomas. Type 1 generalized diffuse: very painful adipose tissue without lipomas. Type 2 generalized nodular: generalized pain in the adipose tissue that is more intense inside and around the lipomas. Type 3 localized nodular: painful adipose tissue exclusively within and around lipomas. Lastly, type 4 juxta-articular: deposition of painful solitary fat in the proximity of large joints [1,7]. This case matched with Dercum's disease type 2 as he is suffering from painful lipomas.

Laboratory analysis lacks specificity. Acute phase reactant values may be raised because adipose tissue inflammation is the defining feature of Dercum's disease. Since there are currently no known biomarkers linked to Dercum's disease, the final diagnosis is determined based on the results of the histology. The histopathology results of tissue specimens are consistent with those of fatty connective tissues and are identical to lipomas [8], as seen in the histopathology results of our patient (Figure 2). Radiological investigations also aid in the detection of subcutaneous nodules [9].

Diagnosis of Dercum's disease can be challenging for doctors at certain times. Fibromyalgia, which can manifest similarly to Dercum's disease, is considered a differential diagnosis. However, adiposis dolorosa mostly causes more widespread and intense pain than fibromyalgia. Moreover, familial multiple lipomatosis, which is an autosomal dominant disease, typically manifests as multiple subcutaneous lipomas of varying sizes that are broadly distributed; however, they do not cause incapacitating pain, as seen in Dercum's disease [10].

As Dercum's disease has the potential to develop into a chronic, progressive, and debilitating condition, it is essential to educate patients about the disease state. There are currently no treatments approved by the US Food and Drug Administration for Dercum's disease, and the effectiveness of treatments that have been attempted is variable. Transdermal lidocaine has been demonstrated to significantly reduce pain by >60%. The regulation of potential sympathetic nervous system hyperactivity causes pain relief [11]. Patients treated with deoxycholic acid experience a reduction in tumor size, as evidenced by radiographs, as well as a significant improvement in symptoms [12]. A spinal cord stimulator showed a significant improvement in burning pain [13]. Pain in our patient relatively improved by using an oral non-opioid pain reliever (acetaminophen).

It is imperative for primary care physicians and dermatologists to possess a comprehensive understanding of Dercum's illness to accurately diagnose and recognize such disorders in outpatient settings. The timely recognition and assessment of individuals is crucial to commence intervention and direct them to the relevant specialized fields, thereby mitigating the negative societal perception associated with their condition and ultimately enhancing their overall well-being. Dercum's disease is seen more frequently in obese women [5]; our patient, however, had a normal BMI and was a young male patient, making it a unique presentation in the literature.

## Conclusions

Diagnosis of Dercum's disease should be considered in generalized painful lumps. Although the disease is commonly seen in obese women, males with a normal BMI are also affected. In general, the recommended clinical protocol for the evaluation of painful masses includes a comprehensive history and physical examination, serologic markers of acute-phase reactants, and biopsy of suspicious lesions.

## References

[1] Hansson E, Svensson H, Brorson H (2012). Review of Dercum's disease and proposal of diagnostic criteria, diagnostic methods, classification and management. Orphanet J Rare Dis.

[2] Dercum FX (1892). Three cases of a hitherto unclassified affection resembling in its grosser aspects obesity, but associated with special nervous symptoms: adiposis dolorosa. Am J Med Sci.

[3] Kucharz EJ, Kopeć-Mędrek M, Kramza J, Chrzanowska M, Kotyla P (2019). Dercum's disease (adiposis dolorosa): a review of clinical presentation and management. Reumatologia.

[4] Rosmaninho A, Pinto-Almeida T, Fernandes IC, Machado S, Selores M (2012). Do you know this syndrome?. An Bras Dermatol.

[5] Lemaitre M, Aubert S, Chevalier B (2021). Rare forms of lipomatosis: Dercum’s disease and Roch-Leri Mesosomatous lipomatosis. J Clin Med.

[6] Al-Ghadban S, Cromer W, Allen M, Ussery C, Badowski M, Harris D, Herbst KL (2019). Dilated blood and lymphatic microvessels, angiogenesis, increased macrophages, and adipocyte hypertrophy in lipedema thigh skin and fat tissue. J Obes.

[7] Molina JD, Nai GA, Andrade TC, Abreu MA (2019). Dercum's disease: a rare and underdiagnosed disease. An Bras Dermatol.

[8] Lam A, Aukerman W, Winegarden B, Morrissey S (2021). Lurking under the surface: Dercum's disease. Cureus.

[9] Mardasi E, Russon A, Mansberg V, Bui C, Mansberg R (2023). Incidental adiposis dolorosa (Dercum's disease) detected on 18 F-DCFPyL PET/CT. Clin Nucl Med.

[10] Li LZ, Kan CFK, Webb-Detiege TA (2021). Differential diagnosis of a case of Dercum's disease with possible familial involvement and review of literature. Yale J Biol Med.

[11] Desai MJ, Siriki R, Wang D (2008). Treatment of pain in Dercum's disease with Lidoderm (lidocaine 5% patch): a case report. Pain Med.

[12] Silence C, Rice SM, Liteplo A (2023). Deoxycholic acid for Dercum disease: repurposing a cosmetic agent to treat a rare disease. Cutis.

[13] Rogowski BC, Bharthi R, Zaki PG (2023). Spinal cord stimulation: a novel approach to pain management in Dercum's disease. Surg Neurol Int.

